# Effects of neurofeedback on major depressive disorder: a systematic review

**DOI:** 10.31744/einstein_journal/2023RW0253

**Published:** 2023-07-06

**Authors:** Isabelly Cristine de Souza Dobbins, Murilo Bastos, Renan Cassiano Ratis, Weber Claúdio Francisco Nunes da Silva, Juliana Sartori Bonini

**Affiliations:** 1 Department of Pharmaceutics Science Universidade Estadual do Centro-Oeste Guarapuava PR Brazil Department of Pharmaceutics Science, Universidade Estadual do Centro-Oeste, Guarapuava, PR, Brazil.

**Keywords:** Depressive disorder, major, Brain waves, Neurofeedback

## Abstract

**Background:**

Major depressive disorder is a difficult-to-treat psychological disorder. Approximately 30% of patients with major depressive disorder do not respond to conventional therapies; thus, the efficacy of alternative therapies for treating major depressive disorder, such as neurofeedback, a non-invasive neuromodulation method used in the treatment of psychiatric diseases, must be investigated.

**Objective:**

We aimed to evaluate the efficacy of neurofeedback in minimizing and treating major depressive disorder and its application as a substitute to or an adjuvant with conventional therapies.

**Methods:**

We searched for experimental studies published between 1962–2021 in Scopus, PubMed, Web of Science, and Embase databases and identified 1,487 studies, among which 13 met the inclusion exclusion criteria.

**Results:**

We noted that not all patients responded to neurofeedback. Based on depression scales, major depressive disorder significantly improved in response to neurofeedback only in a few individuals. Additionally, the number of training sessions did not influence the results.

**Conclusion:**

Neurofeedback can reduce depression symptoms in patients; however, not all patients respond to the treatment. Therefore, further studies must be conducted to validate the effectiveness of neurofeedback in treating major depressive disorder.

## INTRODUCTION

Unipolar depression or major depressive disorder (MDD) is a psychological disease that has become increasingly common over the years; the Pan American Health Organization^(1)^ has reported that this disease occurs worldwide and mostly affects the ability of individuals to perform daily activities. In Brazil, 61% of the population suffers from depression, a rate higher than the global average in terms of mental health disorders.^(2)^Additionally, after the coronavirus disease 2019 (COVID-19) pandemic, the incidence of mental health disorders has increased worldwide, particularly in Latin America.^(2)^

Psychotherapeutic treatments include supportive psychotherapy and psychoanalytic, interpersonal, cognitive, and behavioral therapy.^(3)^ Drugs used to treat depression include tricyclic antidepressants monoamine oxidase inhibitors, and psychomotor stimulants; however, the efficacy of these drugs in depressive disorders remains unclear.^(3)^ Furthermore, approximately 30% of patients do not respond to these psychotherapy or drug interventions. Thus, exploring new interventions for depression with better efficacy is imperative. In this regard, neurofeedback (NF) has been proven an appropriate therapy for MDD.^4^

Neurofeedback is a technique widely used in the last decades to primarily treat psychiatric diseases. Neurofeedback leverages the ability of the patients to learn to control and balance their cerebral activities using brain waves in specific regions of interest (ROIs).^(7,8)^

During training sessions, patients receive feedback (audible or visual) about their mental state and are subsequently requested to attempt modifying the presented results.^(9)^ This impulse usually focuses on the decision-making of patients with depression, with attention paid to the cost-benefit performance and language used.^(10)^ Thus, NF can successfully modify these waves without the need to constantly sedate the patients. After the patients receive the feedback and try to regulate the executed neural activity, they can improve their condition, notably depression and clinical symptoms.^(7,11)^

As the treatment constitutes a non-invasive neuromodulation mechanism, it can be used as a replacement or complement to the existing treatments. Thus, the effectiveness of NF in the treatment of psychiatric illnesses, including MDD, has been extensively studied.^(4,5,7)^ Considering the relevance of non-pharmacological interventions in MDD, we systematically reviewed the literature on using NF as a non-drug treatment for MDD, either as a substitute or an adjuvant integrated with conventional treatment methods. Furthermore, although NF is a well-established technique, its potential as a conventional method for treating depression remains unelucidated. Therefore, we analyzed the efficacy of NF in treating MDD and improving the health of patients.

## OBJECTIVE

We aimed to evaluate whether neurofeedback is an effective intervention for major depression and whether it can substitute conventional psychotherapy and drug interventions or be integrated with these methods.

## METHODS

### Research strategy and screening of articles

This review follows the PRISMA checklist.^(12)^ Reports on experimental studies conducted between 1962 and April 19, 2021, and published in English, were searched and identified from the Scopus, PubMed, Web of Science, and Embase databases. The MeSH terms “Neurofeedback” and “Depressive Disorder, Major” were used as search strings on MedLine (PubMed). The page displayed their synonyms when redirected to their respective links; these synonyms were verified, and all cited synonyms were copied. After using all these terms, we assessed whether we should include more terms. We used the following descriptors by means of Boolean operators “OR” and “AND”: (“Neurofeedbacks” OR “Brainwave Biofeedback” OR “Biofeedback, Brainwave” OR “Biofeedbacks, Brainwave” OR “Brainwave Biofeedbacks” OR “Alpha Feedback” OR “Alpha Feedbacks” OR “Feedback, Alpha” OR “Feedbacks, Alpha” OR “Electromyography Feedback” OR “EEG Feedback” OR “EEG Feedbacks” OR “Feedback, EEG” OR “Feedbacks, EEG” OR “Electroencephalography Biofeedback” OR “Biofeedback, Electroencephalography” OR “Biofeedbacks, Electroencephalography” OR “Electroencephalography Biofeedbacks” OR “Alpha Biofeedback” OR “Alpha Biofeedbacks” OR “Biofeedback, Alpha” OR “Biofeedbacks, Alpha” OR “Brainwave Feedback” OR “Brainwave Feedbacks” OR “Feedback, Brainwave” OR “Feedbacks, Brainwave” OR “Neurofeedback” (MeSH Terms)) AND (“Depressive Disorders, Major” OR “Major Depressive Disorders” OR “Major Depressive Disorder” OR “Paraphrenia, Involutional” OR “Involutional Paraphrenia” OR “Involutional Paraphrenias” OR “Paraphrenias, Involutional” OR “Psychosis, Involutional” OR “Involutional Psychoses” OR “Involutional Psychosis” OR “Psychoses, Involutional” OR “Depression, Involutional” OR “Involutional Depression” OR “Melancholia, Involutional” OR “Involutional Melancholia” OR “Depressive Disorder, Major” [MeSH Terms]).

### Selection criteria

Using the MeSH terms “Neurofeedback” and “Major Depressive Disorder,” along with the text word search, we identified 1,487 studies, which were then deposited on Mendeley^®^ with the automatic exclusion of some articles. In total, 1,293 studies were selected for the next screening step.

Subsequently the articles were imported into the Rayyan app^(13)^ to examine whether they met the inclusion/exclusion criteria. Two authors selected the studies from a spreadsheet containing the following information article title, author names, year of publication, intervention tool, main result, included or excluded, and justification for selecting the articles. After reading the titles and abstracts, 96 articles were selected for subsequent analysis. Two reviewers read the complete articles; a third reviewer was called in case of any disagreements.

The inclusion criteria were studies that investigated the use of NF as a substitute treatment or as a secondary therapy for patients with MDD of any age and validated its efficacy in decreasing the MDD levels in scales that indicate depression stages and symptoms.

The exclusion criteria were studies that: used other intervention types measuring only the results with electroencephalography (EEG) without any NF training; investigated NF techniques; used animals; included patients with post-traumatic stress; analyzed other types of depression; did not use depression scales; evaluated only cognitive ability or waves as biomarkers, and did not use NF as a treatment or rehabilitation for patients with MDD.

### Data extraction and analysis

The data were extracted on an Excel spreadsheet, with the following topics for each article: publication date, sample characteristics, control and experimental group, type and duration, variable, pre- and post-intervention measurements of each group using depression scales, and the results that each study obtained.

## RESULTS

**Figure 1:**
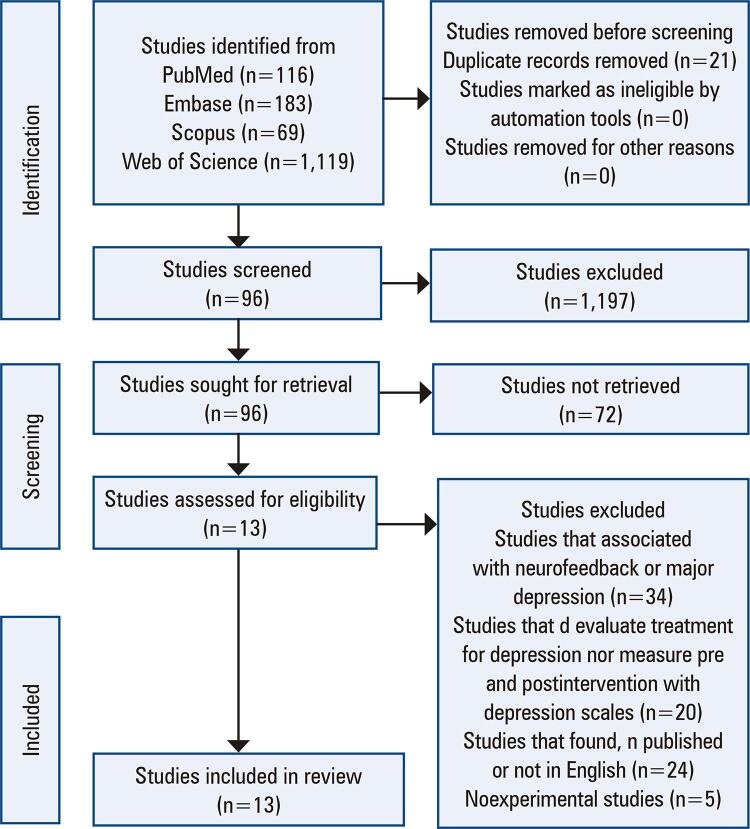
Flow chart illustrating the phases for searching relevant studies and the selection criteria of the studies

Among the articles analyzed, 13 were eligible for this review (Figure 1).

We included 13 experimental studies (Table 1),^(4-7,14-22)^ among which five used asymmetry protocol,^(6,7,14,15,22)^ two used real-time functional magnetic resonance imaging (rtfMRI)-EEG-NF,^(16,17)^ four utilized rtfMRI-NF,^(5,18-20)^ one used fMRI-NF,^(4)^ and one used slow cortical potential NF (SCP-NF).^(21)^ Additionally, not only the protocols but the number of training sessions used were also highly variable among the studies (Table 1).

**Table 1:** Characteristics of the studies included in the systematic review

Study	NF type	Methodology	Depression scales	Total number of sessions	Results
Mehler et al.^(4)^	fMRI-NF	Any mental strategy NFE: imagery of positive stimuli NFS: imagery of scenes	HDRS, HADS	5	Significant improvement up to 25% upregulation of emotion areas and 50% upregulation of a control region activated by visual scenes
Tsuchiyagaito et al.^(5)^	rtfMRI-NF	Upregulated LA activity with kynurenine measured using two task block regressors, “happy” and “count”	MADRS	2	Thirteen participants exhibited a ≥25% reduction in the MADRS score
Cheon et al.^(6)^	Asymmetry rotocol	Training of the left hemisphere with beta waves at F3 and with alpha/theta waves at Pz	HAM-D, HAM-A, BDI-II, BAI	16 to 24	Significant improvement in HAM-D scores
Chen et al.^(7)^	Asymmetry protocol	Training with delta, theta, alpha, and low and high beta waves at P3 and P4	BDI-II, BAI	10	Significant improvement of cognitive depression but not somatic depression
Wang et al.^(14)^	Asymmetry protocol	ALAY protocol, down-training of the alpha power at F3, and up-training of alpha power at F4	BDI-II, BAI	6	57.14% of the subjects did not respond to the intervention
Choi et al.^(15)^	Asymmetry protocol	EEG from F3 and F4 in a trial with classical music where the participants had to keep the sound on and try to raise its volume	HAM-D, BDI-II	50% of the participants with depression showed significant improvement	50% of the participants with depression showed significant improvement
Zotev et al.^(16)^	rtfMRI-EEG-NF	Real-time display screen for happy memories, with task runs and simultaneously frontal EEG in the upper alpha band	HDRS, HARS, MADRS, SHAPS, HAS, TAS, POMS, STAI, VAS	2	Significant improvement by VAS, POMS, HDRS, and SHAPS
Zotev et al.^(17)^	rtfMRI-EEG-NF	Real-time GUI display screen for happy memories with task runs alpha and high-beta EEG for F3 and F4	MADRS, POMS, STAI, SHAPS	Differences between the control and experimental groups did not reach significance	The control and experimental group were not significantly different
Takamura et al.^(18)^	rtfMRI-NF	A run of rest block (gray) and up-regulation block (green) that the participants had to raise the signal in the green as compared to the gray signal, target ROI	BDI-II, HDRS, RRQ	5	Significant improvement by RRQ
Young et al.^(19)^	rtfMRI-NF	Real-time display screen to recall happy autobiographical memories with task runs, target ROI	MADRS, POMS, VAS, STAI	1	Significant improvement in the scales related to mood effects
Young et al.^(20)^	rtfMRI-NF	Patients had to retrieve positive memories while attempting to increase the hemodynamic activity in the assigned region. With task runs.	MADRS, BDI-II, SHAPS, HAM-D, HAM-A, BDI-II, SHPS	2	The responder groups exhibited significant improvement
Schneider et al.^(21)^	SCP-NF	Trial of visual feedback of SCP where the assignment of trial type (negative or positive slow potential) was discriminated by the stimuli	HAM-D, GAS, BPRS	20	Minimal correlation with depression scales
Liu^(22)^	Asymmetry protocol	Alpha-beta protocol (suppress alpha wave and amplified beta wave) of the left hemisphere at F3 and two other electrodes on the eardrum	BDI-II	9	No significant improvement

fMRI-NF: Functional magnetic resonance imaging neurofeedback; HDRS/HAM-D: Hamilton Depression Rating Scale; HADS/HAS: Hospital Anxiety and Depression Scale; MADRS: Montgomery–Asberg Depression Rating Scale; HAM-A: Hamilton Anxiety Rating Scale; BDI-II: Beck Depression Inventory; BAI: Beck Anxiety Inventory; SCP-NF: Slow cortical potential Neurofeedback; GAS: Goal Attainment Scaling; BPRS: Brief Psychiatric Rating Scale; POMS: Profile of Mood States; VAS: Visual Analogue Scale; STAI: State-Trait Anxiety Inventory; RRQ: Rumination-Reflection Questionnaire; EEG: electroencephalography; SHAPS: Snaith–Hamilton Pleasure Scale; TAS: Toronto Alexithymia Scale; GUI: graphical user interface; ROI: region of interest.

Young et al.^(19)^conducted one training session for amygdala activity in a control and an experimental group and observed that the results did not differ between the two groups. The Profile of Mood States (POMS) depression scores were not significantly different between the groups; however, the scales related to mood effects (such as Visual Analogue Scale [VAS] happiness and POMS anger) were significantly reduced in the experimental group.

Moreover, asymmetry protocol did not appear to influence the results. Choi et al.^(15)^ included 12 participants in the experimental group, and only 50% of the participants showed an increase in depression symptoms. Similarly, in the study conducted by Wang et al.,^(14)^ four of seven patients (57.14%) were non-responders. Liu^(22)^ observed that compared with the control group, the experimental group exhibited an increased recurrence of symptoms, but this effect was not significant. Chen et al.^(7)^ observe a change in wave amplitudes in the participants and demonstrated that emotional symptoms improved as the high beta amplitude decreased. However, they did not observe a significant correlation between Beck Anxiety Inventory (BAI) and Beck Depression Inventory (BDI-II). Among these five studies, only the study by Cheon et al.^(6)^ reported a significant improvement in cumulative remission rates by Hamilton Depression Rating Scale (HAM-D/HDRS) (55%), with reduced anxiety symptoms and severity of clinical illness.

Studies using the rtfMRI-NF protocol have not demonstrated significant correlations between the rtfMRI-NF performance and depression scales. Although Takamura et al.^(18)^ successfully regulated the ROI, only rumination was correlated to this regulation, not the improvement in BDI-II and HDRS, and the Hamilton Anxiety Rating Scale (HAM-A) scores did not significantly differ between the groups at any visit. The results of Young et al.^(20)^ were similar to those of Wang et al.^(14)^ and Choi et al.;^(15)^ twelve of nineteen participants responded to NF with a 50% decrease in the Montgomery–Asberg Depression Rating Scale (MADRS) score, demonstrating that the change was significant for only the responders Tsuchiyagaito et al.^(5)^reported that only 13 participants responded to the treatment, indicating a ≥25% reduction in MADRS scores.

Mehler et al.^(4)^ used the fMRI-NF protocol without real time on two experimental groups, the NFE group that was asked to upregulate the emotion areas and the NFS group that had to upregulate the areas activated by visual scenes. The study lacked a control group. The NFE and NSF groups improved by 42% and 44%, respectively, on the HDRS-17. However, despite stimulating a rewarding experience,only 25% (4/16) and 50% (8/16) of the NFE and NFS participants, respectively, responded to the treatment.

Finally, only Zotev et al.^(16,17)^ used rtfMRI-EEG-NF. They measured emotional states in VAS POMS. HDRS and Snaith–Hamilton Pleasure Scale (SHAPS) were significantly improved; however, the other scales were unaffected. However, Zotev et al.^(17)^ observed no significant difference between the control and experimental groups.

### Quality assessment

The 13 studies included in this systematic review were assessed with the PEDro scale for methodological quality (Table 2). Six of these studies^(7,14-17,20)^had a score ≥5, demonstrating their moderate to high methodological quality. The remaining^(4-6,18,19,21,22)^ had a score ≤4, demonstrating their low methodological quality. All studies, except those by Cheon et al.^(6)^ and Takamura et al.,^(18)^ included statistical comparisons between groups. Furthermore, five studies^(4-7,18)^ did not have a control group and compared only experimental groups. Only one study^(19)^ blinded the subjects, and two^(4,20)^ blinded the therapists and assessors.

**Table 2:** Quality assessment of the studies using the PEDro scale

Study	Eligibility criteria	Subjects randomly allocated	Concealed allocation	Groups similar at baseline	Blinding of subjects	Blinding of therapists	Blinding of assessors	<15% dropout	intention to treat analysis if necessary	between-group statistical comparisons	Point estimate and variability	Total
Mehler et al.^(4)^	Yes	Yes	No	Yes	No	Yes	Yes	No	No	Yes	Yes	2/10
Tsuchiyagaito et al.^(5)^	Yes	No	No	No	No	No	No	Yes	Yes	Yes	No	3/10
Cheon et al.^(6)^	Yes	No	No	No	No	No	No	Yes	Yes	No	Yes	3/10
Chen et al.^(7)^	Yes	No	No	Yes	No	No	No	Yes	Yes	Yes	Yes	5/10
Wang et al.^(14)^	No	Yes	No	No	No	No	No	No	No	Yes	Yes	6/10
Choi et al.^(15)^	No	No	No	No	No	No	No	Yes	Yes	Yes	Yes	6/10
Zotev et al.^(16)^	Yes	Yes	No	Yes	No	No	No	Yes	Yes	Yes	Yes	5/10
Zotev et al.^(17)^	Yes	No	No	No	No	No	No	No	No	Yes	No	5/10
Takamura et al.^(18)^	Yes	No	No	No	No	No	No	Yes	No	Yes	Yes	2/10
Young et al.^(19)^	Yes	No	No	No	No	No	No	Yes	Yes	No	No	3/10
Young et al.^(20)^	No	No	No	Yes	No	No	No	Yes	Yes	Yes	Yes	9/10
Schneider et al.^(21)^	Yes	Yes	Yes	Yes	Yes	Yes	Yes	Yes	no	Yes	Yes	4/10
Liu^(22)^	No	No	No	Yes	No	No	No	Yes	Yes	Yes	Yes	3/10

## DISCUSSION

We have systematically reviewed the reports on NF as a treatment tool for MDD. In these studies, NF was used with functional magnetic resonance, EEG, and asymmetry protocol, as alpha, beta, and theta bands.

The plasticity of brain function appears to be associated with tasks linked mainly to autobiographical memory, as demonstrated by Young et al.^(19)^ Linden^(9)^ also observed that patients could readily recollect positive thoughts about themselves, consequently exhibit better improvement. However, this was only observed with the responder groups, and the disparity in response to NF remains unclear. Five^(4,5,14,15,20)^ of the studies had a certain number of non-responders. Conversely, Schneider et al.^(21)^ conducted 20 sessions for the experimental group, demonstrating significant improvements in the first five sessions, with minimal correlation with HAM-D, Goal Attainment Scaling, and Brief Psychiatric Rating Scale, but the remaining 15 sessions did not have any effect on alleviating MDD. This may be because the reward given to the patients who completed the session positively motivated them, but this decreases the effectiveness of the treatment.

To emphasize the efficacy of the asymmetry protocol, we can take the example of Liu et al.,^(22)^ who used the NF training program as an audiovisual intervention. The experimental group did not show many changes: the recurrence of symptoms was stable, and the regulation of brain waves was altered. The focus of this research was that the state of the intensity of depression did not worsen; however, a decrease in intensity was also not observed. Peeters et al.^(8)^ and Liu^(22)^ reported that NF appears to prevent higher levels of depression intensity, preventing the recurrence of symptoms. However, the lack of improvement observed by Peeters et al.^(8)^ could be attributed to the limited number of sessions. However, Wang et al.^(14)^ stated that the efficacy of NF is not influenced by the number of training sessions but by whether excellence was achieved.

However, Cheon et al.^(6)^demonstrated a significant improvement in depression using HAM-A, HAM-D, BDI and BAI scales, with the training of beta at F3 and alpha and theta at Pz, twice or three times a week for eight weeks, which is 16–24 sessions of 30 minutes each, improving cumulative rates in HAM-D by 55%. Thus asymmetry protocol can achieve diverse results.

Meanwhile, rtfMRI-NF appears to already follow a system, as revealed by Young et al.^(19,20)^ and Zotev et al.,^(16,17)^ who used training sessions that alternated between rest, happy memories, and count.

In the study by Young et al.,^(19)^ patients were asked to recall their happy memories as an intervention. The authors conducted seven sessions of NF, each lasting 8 minutes and 40 seconds. Each session had three rounds, one for practice, one for rest, and one to transfer the data to analyze if the patient can control this thought without NF. The practice was divided into three parts, alternating between rest, happy memories, and count (the patient counted backward after a supposed whole number). The scales of MDD were not affected by this method; however, an increase in VAS was observed, indicating that rtfMRI-NF could improve the mood of the participants. However, Young et al.^(20)^focused on ROIs related to memory recovery using the same intervention with one less session and still achieved significant improvement; however, as mentioned already, this improvement was only observed for 12 of the 19 participants in the experimental group.

Zotev et al.^(17)^ used the same intervention as Young et al.,^(20)^with the same number of sessions. The VAS, POMS, HDRS, and SHAPS scales used by Zotev et al.,^(17)^ as well as the ROI that revolves around emotional regulation, significantly improved. However, Zotev et al.^(23)^ used the same intervention with anxiety, depression, and anhedonia scales and other ROIs with specific waves in certain regions and observed that depression symptoms were mitigated and motivation increased with decreasing anxiety, but the differences between the placebo and experimental groups were not significant.

Although real-time NF and EEG-NF have their differences, combining these two methods using specific cerebral regions and alpha and beta bands may have a large potential to reduce anhedonia and comorbid anxiety and even serve as a suitable treatment for MDD.^(17,23)^ Thus, the role of NF in treating MDD should be further investigated. This review indicated that NF could greatly improve mood, emotions, and the recurrence of depression symptoms in patients; however, somatic depression seems to have a minimal correlation with NF, considering the scales analyzed by these studies.

### Limitations

We observed three limitations in the studies analyzed in this review: lack of patterns, limited sample size, and variable number of sessions among the studies. First, lack of pattern refers to the lack of a specific sound and image, a consistent wave frequency, or a unique method used in these studies. We lack clarity regarding the precise pattern, which is ideal for treating the patient. Moreover, the amount of sessions required for successful treatment has not been confirmed; notably, the persistence of the NF effects after terminating the treatment must be investigated. Second, the sample sizes of the studies were small, with <50 patients with MDD in the experimental group, affecting the reliability of the results. Furthermore, patients with critical depression were excluded from the studies, leading us to believe that NF treatment may not be sufficient for complex cases.

## CONCLUSION

Patients with major depressive disorder, a recurrent psychological disease, rarely respond to psychotherapy or drug therapy. Thus, alternative treatments, such as neurofeedback, have recently gained importance in treating major depressive disorder. Most studies included in this review suggested that neurofeedback improved major depressive disorder and its symptoms in patients. However, the findings of these studies are insufficient to establish neurofeedback as a new therapy for major depressive disorder with 100% efficacy.

Neurofeedback possibly improves abilities and acts on patients who have already received other treatments for depression but have not experienced any improvements. However, it remains to be determined whether neurofeedback acts as a primary or secondary therapy for depression. Therefore, the role of neurofeedback in treating depression requires further investigation.
